# Midbrain Frequency Representation following Moderately Intense Neonatal Sound Exposure in a Precocious Animal Model (*Chinchilla laniger*)

**DOI:** 10.1155/2016/3734646

**Published:** 2016-11-08

**Authors:** Lisa M. D'Alessandro, Robert V. Harrison

**Affiliations:** ^1^Department of Physiology, University of Toronto, Toronto, Canada M5S 1A8; ^2^Institute of Biomaterials and Biomedical Engineering, University of Toronto, Toronto, Canada M5S 3G9; ^3^The Auditory Science Laboratory, Program in Neurosciences and Mental Health, The Hospital for Sick Children, Toronto, Canada M5G 1X8; ^4^Department of Otolaryngology–Head and Neck Surgery, University of Toronto, Toronto, Canada M5G 2N2

## Abstract

Auditory brain areas undergo reorganization resulting from abnormal sensory input during early postnatal development. This is evident from studies at the cortical level but it remains unclear whether there is reorganization in the auditory midbrain in a species similar to the human, that is, with early hearing onset. We have explored midbrain plasticity in the chinchilla, a precocious species that matches the human in terms of hearing development. Neonatal chinchillas were chronically exposed to a 2 kHz narrowband sound at 70 dB SPL for 4 weeks. Tonotopic maps in inferior colliculus (central nucleus) were defined based on single neuron characteristic frequency. We hypothesized an overrepresentation of the 2 kHz region of the maps. However, we observed a significant decrease in the proportion of neurons dedicated to the 2 kHz octave band and also away from the exposure frequency at 8 kHz. In addition, we report a significant increase in low frequency representation (<1 kHz), again a change to tonotopic mapping distant to the 2 kHz region. Thus in a precocious species, tonotopic maps in auditory midbrain are altered following abnormal stimulation during development. However, these changes are more complex than the overrepresentation of exposure related frequency regions that are often reported.

## 1. Introduction

The maturity of the auditory system at birth differs between species. Many common laboratory species, such as the mouse or rat, are altricious, that is, born with a relatively immature auditory system. In such animals, final developmental maturation at the cochlear level occurs postnatally (e.g., [1–4]) and the onset of cochlear function occurs many days after birth, for example, on postnatal day 12 (P12) in rats [5, 6]. Humans, on the other hand, are relatively precocious. At birth, the cochlea is well-developed [7] and there is clear evidence for auditory responses* in utero* [8, 9]. An appropriate animal model for studies relating to human auditory development is a precocious one such as the chinchilla. The cochlea of the newborn chinchilla is structurally and functionally mature [10]. Tonotopic maps in primary auditory cortex and secondary auditory cortical fields are well-ordered and neurons are sharply tuned by P3 [11]. Furthermore, unlike common laboratory species (cat, rat, guinea pig), chinchilla audibility curves more closely resemble those of humans across a broad range of frequencies [12, 13]. Because the state of the chinchilla auditory system, at birth, is similar to that of a human, this species is an appropriate animal model for studies of neonatal auditory neuroplasticity.

Many studies describing auditory developmental plasticity have revealed alterations in cortical representation in response to enhanced peripheral input [14–17]. One important question that arises is whether this reorganization is intrinsically cortical, or whether it reflects, wholly or partially, reorganization at lower levels of the auditory pathway. Relatively normal thalamo-cortical projection patterns were observed following neonatal deafening in the cat [18], suggesting that cortical changes are reflected at thalamic levels. At the level of the midbrain (inferior colliculus, IC) there is evidence for neural reorganization in response to neonatal cochlear lesions in the (precocious) chinchilla [19]. To the best of our knowledge, experiments in which midbrain plasticity has been reported in response to early sound augmentation have been in altricious species (e.g., mouse: [20]; rat: [21]) where neonatal manipulations are carried out very early in auditory system development. It remains unclear whether in a precocious animal model, an enhanced acoustic environment has any neuroplastic effect at the level of the auditory midbrain.

Our working hypothesis is that the development of neural connections within the ascending auditory pathway is influenced, in large part, by patterns of sensory activity elicited by environmental sound stimulation during an early postnatal period. Experimentally, we hypothesize that passive neonatal exposure to an acoustic frequency-enhanced environment alters the neural representation of sound frequency in the central nucleus of IC compared with age-matched controls. To test this hypothesis, neonatal chinchillas (*Chinchilla laniger*) were exposed for 4 weeks to a moderately intense (70 ± 5 dB SPL), narrowband-enriched (2 ± 0.25 kHz) sound environment. We probed changes in midbrain frequency representation using micro-electrode recordings of neural activity patterns throughout the central nucleus of IC. The sound-exposure stimulus was designed to elicit enhanced neuronal activation, but not to damage cochlear hair cells. To verify this, we measured auditory brainstem evoked response threshold assessments to tonal stimuli (ABR audiograms) at tone frequencies around the sound-exposure stimulus. In addition, we assessed hair cell morphology using scanning electron microscopy, particularly around the 2 kHz cochlear region. These experiments are the first to explore possible neuroplastic effects of neonatal sound exposure at the midbrain level in a precocious species.

## 2. Materials and Methods

### 2.1. Experimental Animals

All procedures were approved by the Animal Care Committee of the Hospital for Sick Children following the Canadian Council for Animal Care guidelines. This study is based on data from fifteen chinchillas (*Chinchilla laniger*; 9 females, 6 males; Roseneath Chinchilla; Roseneath, Ontario, Canada). Eight subjects served as controls, and 7 subjects were exposed, as neonates, to an enhanced acoustic environment (described in Section 2.2 below). In overview, newborn chinchilla pups were chronically exposed to an acoustically enhanced environment for a period of 4 weeks before electrophysiological study of ABR thresholds and neural activity patterns in IC. Subjects were aged between postnatal day 29 (P29) to P34 (140–250 g) at the time of microelectrode recording from central nucleus of IC. Age-matched, nonexposed animals served as experimental controls. Evaluation of cochlear hair cell integrity was made in a subgroup of animals using scanning electron microscopy.

### 2.2. Neonatal Sound-Exposure

We generated a shaped (20 ms rise/fall time; 500 ms on, 1 s off) 2 ± 0.25 kHz narrow-band stimulus (Adobe Audition 2.0, San Jose, CA, USA). The stimulus was continuously presented in free-field (Sony Micro Hi-Fi, CMT BX20i, coupled to Sony transducer Model #SS-CBX20, Minato, Tokyo, Japan), for at least 4 weeks (28–33 days), beginning on postnatal day 0 (P0) or P1. The sound-exposure stimulus was calibrated to be 70 ± 5 dB SPL at the level of the animals' ears. Measurements made at multiple locations in each cage indicated <5 dB variations. The ambient sound spectrum measured in the animal housing enclosure was relatively flat with no significant peaks. During sound exposure, animals did not exhibit abnormal behaviour and appeared to feed normally. There was no significant difference in weight between control and sound-exposed subjects (reported as mean ± SD; controls: 168.3 ± 35.6 g; sound-exposed: 170.4 ± 33.9 g; *p* = 0.77, *t*-test).

### 2.3. Auditory Brainstem Evoked Responses (ABR)

In sound-exposed and control animals, ABR thresholds to broadband (47-*μ*s) clicks (*n* = 50  and 25 for controls and sound-exposed, resp.) and tonal stimuli (1, 1.5, 2, 2.5, 3, 4, and 8 kHz; 4 ms; Blackman envelope; *n* = 26  and 15 for controls and sound-exposed, resp.) were recorded (Smart EP, Intelligent Hearing Systems, Miami, FL, USA). Animals were anaesthetized with ketamine (15 mg/kg, I.P.) and xylazine (2.5 mg/kg, I.P.). Skin needle electrodes were in a mastoid (bulla) vertex configuration. Stimuli were presented monaurally (right ear) through an insert earphone (ER-2, Etymotic Research, Elk Grove Village, IL, USA) in 10 dB intensity steps. ABR signals were based on 512 (tone-pip) or 1024 (click) averages. Threshold was taken as the level at which predominant ABR peaks were just discernible. Subjects were first screened using click stimuli and then tested with tonal stimuli and included in the study if click thresholds were less than 30 dB SPL. Comparison of tonal ABR audiograms in exposed versus control animals is reported in Section 3.1.1.

### 2.4. Microelectrode Recordings in Inferior Colliculus

Animals were anaesthetized with I.P. administration of ketamine (15 mg/kg) and xylazine (2.5 mg/kg). For long term maintenance, subjects were given one-half doses every hour for the duration of data collection. Body temperature was monitored with a rectal probe and maintained thermostatically at 37°C. A total of 11 animals (8 females, 3 males) were used; 6 subjects were controls, and 5 subjects were sound-exposed.

**Figure 1:**
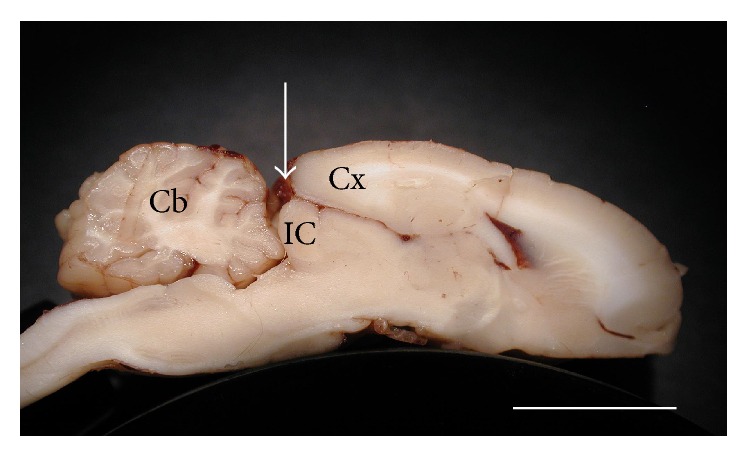
Following surgery the inferior colliculus (IC) is visible for direct dorsoventral electrode placement (indicated by arrow), while the integrity of cortex (Cx) and cerebellum (Cb) are maintained. Sagittal sections through IC were obtained upon completion of recordings. Scale bar indicates 1 cm.

We developed a technique to access the IC that preserves the integrity of the overlying cortex and cerebellum. Following tracheotomy and intubation, the cranium was opened above the junction of the occipital lobe and the cerebellum, and the dura reflected. Surface vessels were cauterized. A flattened, surgical-grade compressed sponge (Otocell® Ear Wicks, Boston Medical Products, Westborough, MA, USA) was gently inserted between occipital cortex and cerebellum. When moistened, this material expands allowing direct visual access of IC for electrode placement as indicated by the arrow in Figure 1. Silicone oil was applied to the preparation to prevent desiccation. This technique was a reliable method of accessing the IC.

Extracellular microelectrode recordings were made on a vibration-isolation table in a sound-attenuating booth (IAC). A remote-control microdrive (MCM Controller Module, FHC, Bowdoin, ME, USA) held two or four high-impedance (2–4 MΩ) tungsten microelectrodes. Electrodes were spaced 0.5 mm apart and were advanced vertically in 50 *μ*m steps to depths of about 3 mm, the approximate dorsoventral extent of the IC, until auditory-responsive neurons were no longer encountered. Electrode track position was confirmed histologically.

Tones were delivered monaurally to the right ear using a high-frequency sound transducer (Intelligent Hearing Systems, Miami, FL, USA) via a short tube and foam ear tip. Recordings were made from the contralateral IC. Stimuli were generated and recordings were stored and analyzed using Tucker Davis Technology (Gainesville, FL, USA) hardware (System 3 components) and software (SigGenRP v. 4.4, BrainWare32 v. 9.19).

Once the IC was visible, electrodes were placed using micro-drive coordinates. A broadband-noise search stimulus (50 ms; 70 dB SPL) was used to detect responsive neurons. Response analysis was made with cos^2^-shaped 50-ms tones presented at 3-4/s. Tones were at frequency intervals of 1/4-octaves from 0.1–0.4 kHz and 1/8-octave spacing from 0.4 to 20 kHz. Stimuli were presented at 4 levels in 10 dB steps starting at low intensities, typically around 0 dB SPL. All stimuli were presented twice; stimulus presentation was varied pseudo randomly.

Electrode signals were amplified, and band-pass filtered (0.3–5 kHz). Action potentials were discriminated online using voltage window thresholding.

### 2.5. Scanning Electron Microscopy (SEM)

Anaesthetized subjects (*n* = 4; 1 female, 3 males) were transcardially perfused with saline (0.9%) followed by cold fixative (2.5% glutaraldehyde in sodium cacodylate buffer, pH 7.4, 4°C). Cochleae were removed, slowly perfused with 2-3 mL of fixative, and then incubated in fresh fixative for 2 hrs. Samples were postfixed for 1.5 hours in 1% osmium tetroxide and dehydrated through graded ethanol incubations. After cochlear dissection, samples were critical-point dried and sputter-coated with gold for SEM imaging. Findings of this study are reported in Section 3.1.2.

## 3. Results

### 3.1. Cochlear Thresholds and Hair Cell Morphology in Control and Sound-Exposed Subjects

The main experimental manipulation in this study was prolonged (4 weeks) exposure of neonatal animals to a moderately intense, narrowband sound stimulus. To verify that this exposure did not cause cochlear threshold elevations or damage to hair cell stereocilia, we assessed cochlear response thresholds using ABR recordings and imaged cochlear sensory epithelium using SEM after sound exposure. We compared results of sound-exposed subjects to age-matched controls, reared without sound-exposure.

#### 3.1.1. ABR Thresholds

**Figure 2:**
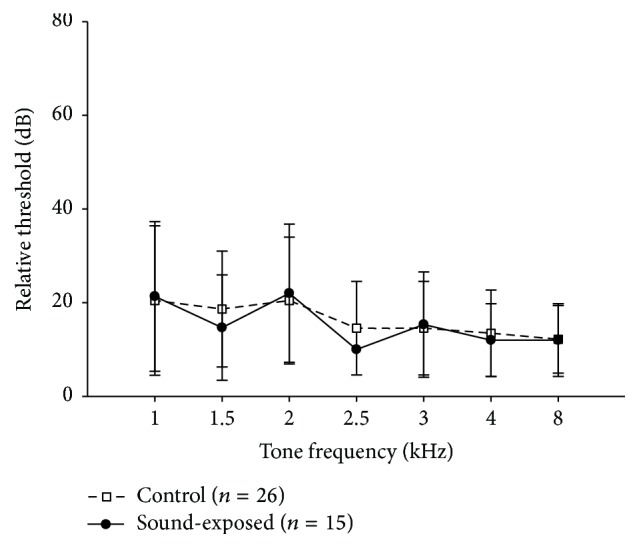
Average ABR thresholds of sound-exposed subjects (filled symbols) at tone-frequencies near the sound-exposure stimulus are not statistically different from those of controls (open symbols). Error bars are ±1 SD.

ABR thresholds to frequency-specific stimuli (1–8 kHz) are plotted for sound-exposed (filled symbol) and control (open symbol) subjects in Figure 2. There is no significant difference between groups (*p* = 0.98, ANOVA), suggesting that the neonatal sound-exposure did not induce changes in cochlear thresholds.

We recorded tone-pip-evoked responses from neurons in inferior colliculus of sound-exposed subjects. We report on several properties of those neurons, relative to control subjects, in Section 3.2, as well as on the tonotopic representation of sound frequency in central nucleus of IC.

#### 3.1.2. Cochlear Imaging with SEM

**Figure 3:**
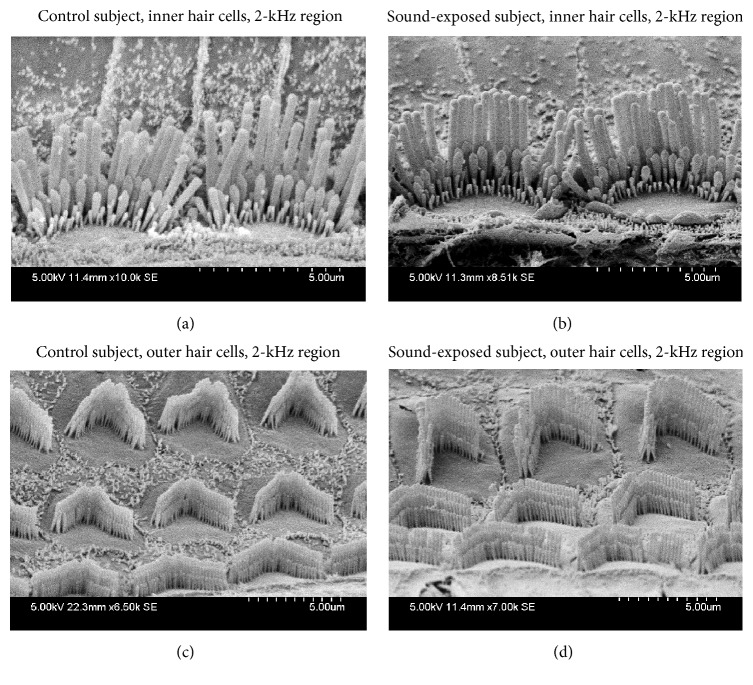
Representative samples of hair cells in the 2 kHz region of the cochlea in control ((a) and (c)) versus neonatally sound-exposed ((b) and (d)) subjects.

Hair cells were imaged along the length of the cochlea with a particular focus on the region corresponding approximately to the 2 kHz sound-exposure stimulus. This SEM analysis was a qualitative study, in which we examined the sensory epithelium for loss of hair cells and any disruption of the stereociliar bundle.  Figure 3 shows representative samples of the sensory epithelium in sound-exposed and control (nonexposed) subjects. We did not detect any signs of unusual morphology in hair cells of sound-exposed subjects. This control study shows that the neonatal acoustic exposure did not result in hair cell or stereocilliar damage as far as can be determined using SEM.

### 3.2. Electrophysiological Responses in IC Neurons

We report here on several response properties of IC neurons in sound-exposed versus control subjects and on the tonotopic representation of sound frequency in the central nucleus of IC (Section 3.2.2).

#### 3.2.1. Response Properties of Neurons in Central Nucleus of IC

We characterized several response properties of IC (central nucleus) neurons and report here minimum thresholds and frequency tuning curve bandwidth 10 dB above threshold (BW_10_). We also qualitatively compared the shape of neural response areas between control and sound-exposed subjects.

**Figure 4:**
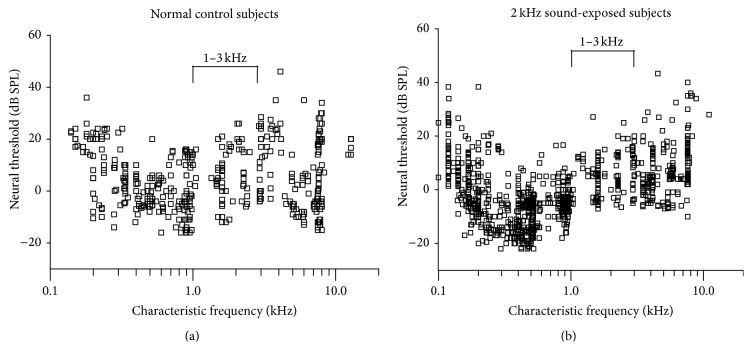
Minimum thresholds of IC neurons in 6 normal controls ((a); *n* = 426 units) and in 5 neonatally sound-exposed animals ((b); *n* = 983 units). There is no difference in neural thresholds near the frequency region (1–3 kHz) of the sound-exposure stimulus.

A comparison of minimum thresholds between sound-exposed and control subjects is shown in Figure 4. Data are from 426 multi-units sampled in 6 control subjects (Figure 4(a)) and from 983 multi-units in 5 neonatally sound-exposed subjects (Figure 4(b)). Near the region of the 2 kHz-centered sound-exposure frequency (1–3 kHz range), there was no difference in neural threshold (reported as average ± standard deviation; controls: 6.7 ± 9.8 dB; sound-exposed: 6.2 ± 7.3 dB, *t*(163) = 0.46, *p* = 0.65). This finding is consistent with our observation of no ABR threshold differences between groups (Section 3.1.1, Figure 2) indicating that neonatal sound exposure did not cause cochlear threshold changes. However, there is one difference between these exposed and control groups: a larger proportion of low-CF neurons in the sound-exposed subjects, which will be quantified in Section 3.2.2 below.

**Figure 5:**
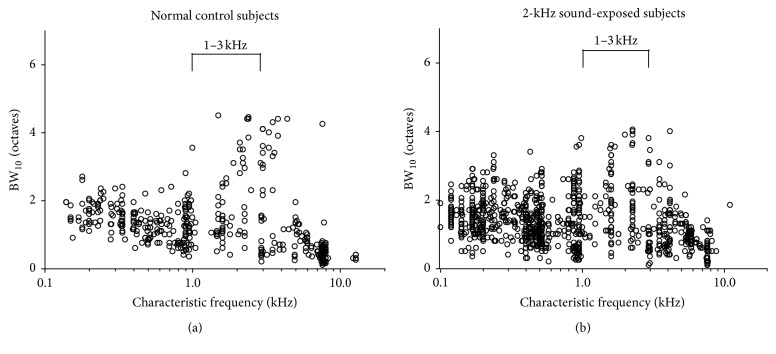
Comparison of frequency tuning bandwidths 10 dB above threshold (BW_10_) in neurons of the central nucleus of IC. There is no difference in BW_10_ near the region of the sound-exposure stimulus (1–3 kHz) in 5 neonatally sound-exposed subjects ((b); *n* = 968 units) compared with 6 controls ((a); *n* = 422 units).

Neural bandwidths 10 dB above threshold (BW_10_), measured in octaves, are plotted as a function of characteristic frequency in Figure 5. Data are from 422 multi-units in 6 control subjects (Figure 5(a)) and 968 multi-units in 5 neonatally sound-exposed subjects (Figure 5(b)). In the frequency region of the sound-exposure stimulus (from 1–3 kHz), there was no significant difference in BW_10_ between groups (reported as average ± standard deviation; controls: 1.7 ± 1.1 octaves; sound-exposed: 1.7 ± 1.0 octaves, *t*(175) = 0.02, *p* = 0.98).

**Figure 6:**
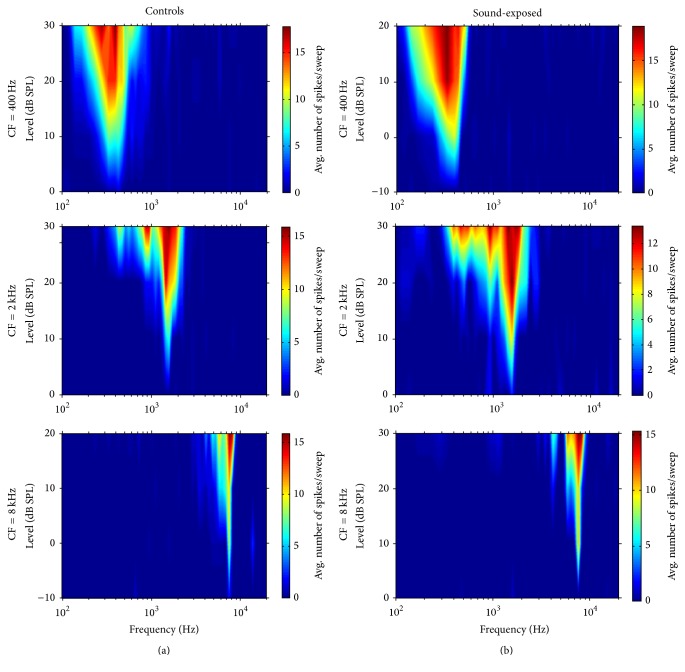
Representative frequency tuning curves in IC for low, mid, and high characteristic frequency (CF) neurons in control (a) and sound-exposed animals (b).

Representative IC tuning curves from low-, mid-, and high-frequency regions are shown in Figure 6 for control (a) and sound-exposed (b) subjects. Qualitatively, tuning curve shape and off-frequency levels of activity were not overtly different between groups over the frequency range from which we recorded (0.1–20 kHz).

#### 3.2.2. Neural Representation of Sound Frequency in IC (Central Nucleus)

**Figure 7:**
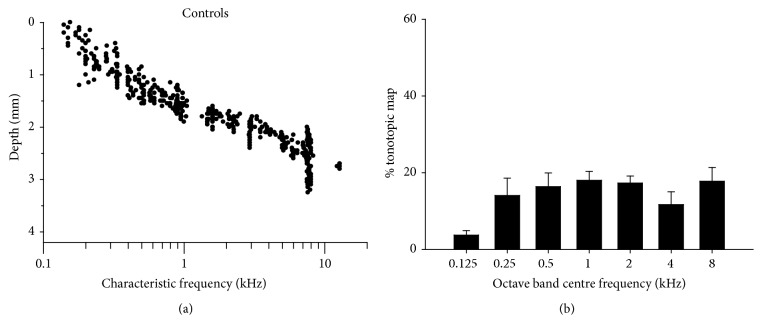
(a) Characteristic frequency (CF) versus dorsoventral IC depth for neurons in normal control subjects. In (b), the histogram indicates the proportion of neurons with CFs in octave-wide frequency bands (centre frequencies as indicated).

The tonotopic organization of neurons in the central nucleus of IC is represented in Figure 7 by plotting CF against electrode depth in the dorsoventral axis of IC (Figure 1). The increase in CF with increasing electrode depth observed in control subjects (Figure 7(a)) is consistent with previous reports [19]. In Figure 7(b), the proportion of recorded neurons within octave bands (with centre frequencies between 0.25–8 kHz) is plotted. Note that CF is quite evenly distributed over the 0.25–8 kHz range of frequencies in these control subjects.

**Figure 8:**
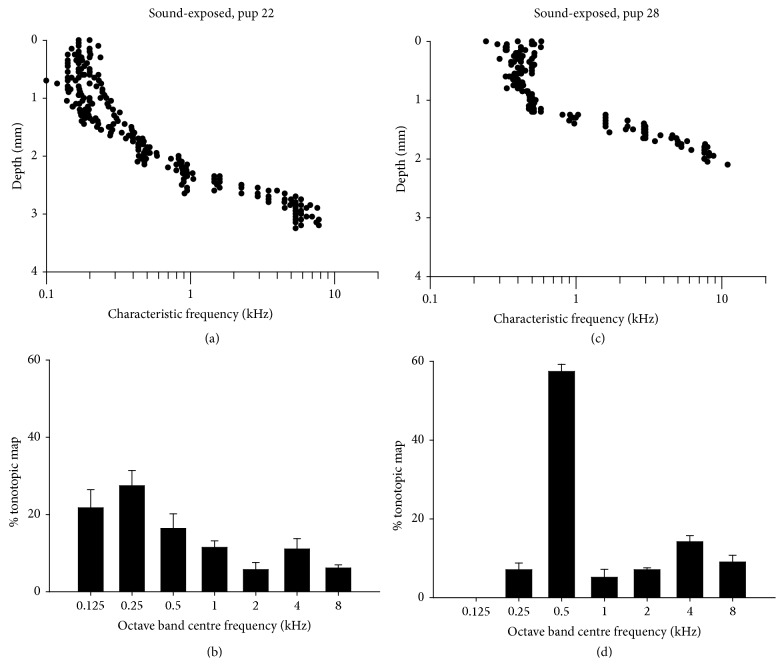
((a) and (c)) Neuron CF versus dorsoventral electrode depth in IC (central nucleus) for two neonatally sound-exposed subjects. In (b) and (d) the proportion of neuron CFs within octave bands (center frequencies 0.125–8 kHz) are plotted.

In neonatally sound-exposed subjects, we observe an increased proportion of neurons tuned to low frequencies, well below the 2 ± 0.25 kHz exposure signal. This is best noted in individual subjects, as shown in Figure 8. Tonotopic maps (CF versus dorsoventral electrode depth) are plotted in Figures 8(a) and 8(c), and the corresponding proportion of neuron CFs within octave bands (centre frequencies 0.125–8 kHz) are plotted in Figures 8(b) and 8(d). Note the overrepresentation of low-CF neurons, and that these overrepresentations are different for each subject. Thus, subject number 22 (Figures 8(a) and 8(b)) has more neurons with CF in the 125–250 Hz range whilst subject number 28 (Figures 8(c) and 8(d)) has overrepresentation near to 500 Hz.

**Figure 9:**
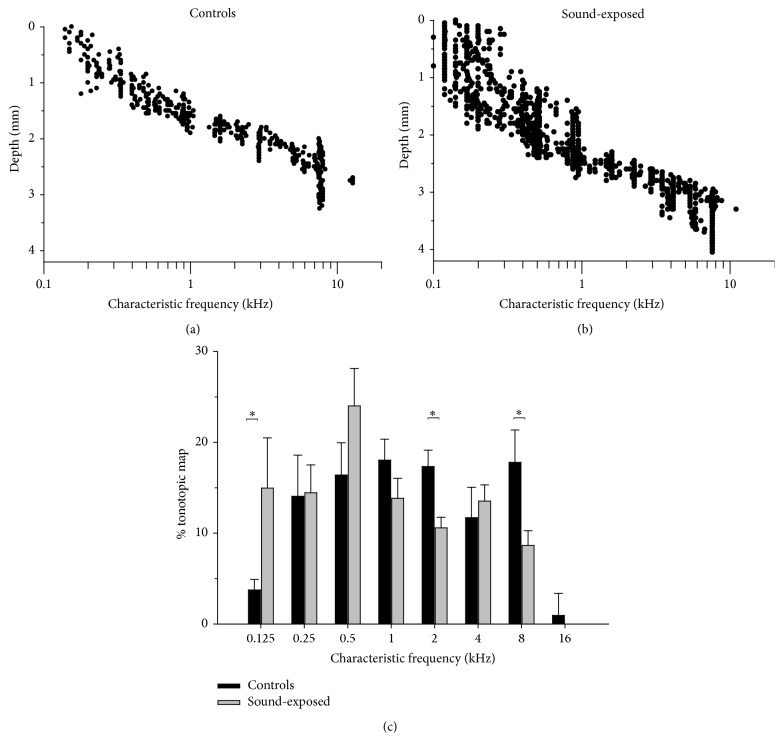
((a) and (b)) Unit characteristic frequency versus electrode depth in IC. Pooled data from 6 normal control subjects ((a); *n* = 426, 16 electrode tracks) and from 5 2 kHz neonatally sound-exposed subjects ((b); *n* = 983, 20 tracks). (c) Comparison of octave-spaced distributions of CF between control and sound-exposed groups ((c); ^*∗*^ *p* < 0.05).

Pooled results from electrode tracks in all subjects are shown in Figure 9. For 6 control subjects (Figure 9(a)), *n* = 426 from 16 electrode tracks. For 5 sound-exposed subjects (Figure 9(b)), *n* = 983 from 20 tracks. As a group, sound-exposed subjects show increased representation of frequencies beginning approximately one octave below the 2 ± 0.25 kHz sound-exposure stimulus. Thus, in general, there is a shift in neural representation of sound frequency towards lower frequencies. This is reflected in the histograms (Figure 9(c)) comparing the octave-spaced distributions of CF between the control and sound-exposed groups. Compared to controls, there is a general increase in frequency representation below 1 kHz for the sound-exposed subjects. Above 1 kHz, this trend is reversed. The significant differences noted in our data are in the octave bands centered at 125 Hz (*t*(7) = 2.39, *p* < 0.05); 2 kHz (*t*(23) = 3.19, *p* < 0.05); and 8 kHz (*t*(14) = 2.72, *p* < 0.05).

#### 3.2.3. Response Properties of IC Neurons in Low-Frequency Regions

The tonotopic distribution data above show a clear increase in proportions of low (<1 kHz) CF neurons in sound-exposed subjects compared to controls. In this regard, it is important to ask whether threshold or tuning characteristics of such low CF neurons differ in the IC of sound-exposed subjects. Whilst in the 2 kHz frequency region near the sound-exposure stimulus we report no significant difference in thresholds (Section 3.2.1, Figure 4), in the frequency region between 0.1 and 1 kHz, neural response thresholds were significantly lower for sound-exposed subjects (reported as average ± standard deviation; −4.3 ± 10.3 dB cf. 2.8 ± 11.2 dB for controls; *t*(311) = 8.0, *p* < 0.001). There was no significant difference in BW_10_ in the frequency region between 0.1 and 1 kHz between groups (reported as average ± standard deviation; controls: 1.4 ± 0.5 octaves; sound-exposed: 1.4 ± 0.6 octaves; *t*(416) = 1.54, *p* = 0.12; Figure 5).

## 4. Discussion

Our study was designed as an animal model to explore the potential effects of abnormal sound exposure in human neonates. Such unusual auditory activation could result from direct exposure of premature babies to high sound levels in NICU environments, or in congenitally deaf infants provided with hearing prostheses (hearing aid or cochlear implant). We therefore selected the chinchilla (*chinchilla laniger*) as our animal model. It is a precocious species and experiences hearing onset* in utero* similar to humans. We chose not to use an altricious species such as the mouse or rat because in such animals cochlear and auditory pathway development at the time of birth is at a significantly earlier stage. For example, hearing onset in the rat occurs between postnatal days 12 to 14 [5]. The state of the chinchilla's peripheral auditory system is similar at birth to that of humans, allowing more confidence in cross-species extrapolation to humans. Our laboratory has many years of experience with this species. We have made extensive recordings in both inferior colliculus (e.g., [19]) and auditory cortex (e.g., [11, 22–24]).

To date, a few studies have examined the effects of an augmented sound environment on properties of neurons in the developing auditory midbrain, and these experiments reveal differing results. In an early study, Moore and Aitkin [25] reported no change in tuning curves or tonotopic organization after exposing newborn kittens to a continuous pure-tone, 8 hours/day for the first 50–75 days of life. Several papers report results from altricious animal models, for example, changes in rat tonotopic maps. Specifically, there is an increase in the proportion of neurons in central nucleus tuned to the exposure frequency: following moderately intense (60–70 dB SPL) 14–16 hr per day exposure to 25 ms tone pips from P9 to P28 [21] and following 12 h/day exposure to continuous pure tones for 3 postnatal weeks [26]. Click-reared subjects (20/sec, 88.5 dB SPL, from P8 to P19–24) exhibit broader tuning curves and no change in spontaneous activity, response latency, or tonotopic maps [27].

More recently, Miyakawa et al. [28] reported a transient narrowing in tuning curve bandwidth following chronic tone-pip exposure (7.5 kHz, 100-ms pip duration, 6 pips in a train at 6 Hz, 1 train every 2 s, 60 dB SPL, from P9 to P25). Long-lasting changes in cortical (but not collicular) tonotopic maps using the same sound-stimulation pattern were observed. A two-tone rearing paradigm (16 + 40 kHz, 80 dB SPL, from P9 to P17, 22-23 hrs/day) revealed large-scale reorganization of tonotopic maps in IC as seen by MRI [20]. These studies have all been made in altricious species with postnatal hearing onset. To the best of our knowledge, the present study is the first to report on the effects of an enhanced acoustic environment on the development of tonotopic maps in inferior colliculus of a precocious animal model.

During development, when there are natural patterns of sensory stimulation, ascending pathways develop normally. However, unusual patterns of stimulus-driven neuronal activity can result in the abnormal development of central sensory maps (e.g., [28, 29]). Both sensory deficits and enhanced sensory environments can be viewed as different-from-normal environments and hence result in abnormal central maps.

### 4.1. Responses of IC Neurons within the 2 ± 0.25 kHz Exposure Frequency Region

At the level of auditory cortex, a number of studies in which cochlear activity patterns have been altered in neonates, either by cochlear lesions or by tonal sound augmentation, report abnormal* overrepresentations* of certain frequency regions related to the border of the cochlear lesion [30] or to the tonal augmentation frequency (e.g., [17]). Interestingly however, in the present study we found a significant* decrease* in neural representation around the sound-exposure stimulus (2 ± 0.25 kHz; see Figure 9(c)) in the inferior colliculus of the chinchilla. This apparent reduction in the population of IC neurons tuned to 2 kHz is not related to any obvious change in neuron response properties, as threshold response and tuning of neurons in this region are not different between control and experimental (sound-exposed) groups (shown in Figures 4 and 5, resp.).

One plausible mechanism for the decrease in neural representation we observed at 2 kHz may relate to homeostatic decreases in synaptic strength following potentiation of neurons in the 2 kHz region. This mechanism has been detailed by Turrigiano and colleagues in cultured rat visual cortex [31]. In the present study, the increased neural firing associated with prolonged exposure to the 2 ± 0.25 kHz sound stimulus may have induced a homeostatic decrease in synaptic strength at those frequencies such that when tones in the region of 2 kHz are subsequently presented to determine frequency response areas, neural responses recorded at the level of IC are decreased. Alternatively, the process may be related to a more peripheral cause. Kujawa and Liberman [32] exposed 16-week-old mice to an octave band of noise (8–16 kHz) for 2 hours at 100 dB SPL. After recovery from a temporary threshold shift, they noted reduced amplitudes of Wave I of ABRs, indicative of a loss of spiral ganglion cells despite normal inner hair cell function. It is possible that the prolonged neonatal sound-exposure in the present study caused some degree of retrograde neural degeneration.

### 4.2. Neural Responses in IC outside the 2 kHz Frequency Region

There were several octave bands in the low-frequency region (125 Hz, 500 Hz; see Figures 8 and 9) where neural representation was increased in sound-exposed subjects. The increase at 125 Hz was significant. In the 4-kHz octave band, and above the 2 kHz sound-exposure region, subjects demonstrated a trend toward greater neural representation. A number of studies have reported on neural array “edge effects” from prolonged sound stimulation and proposed that constant driven activity in a neural frequency region may lead to a release from lateral inhibition (or disinhibition) of neurons neighbouring the sound-exposure stimulus. This can result in an increase in representation of frequencies at the “edges” of the sound exposure stimulus. Eggermont and colleagues have shown this edge effect in cat auditory cortex after relatively moderate levels of sound exposure ([16, 33, 34]; review: [35]).

A critical examination of the literature suggests that increased cortical and subcortical (inferior colliculus) neural responses dedicated to frequencies below the exposure frequency are not uncommon following postnatal rearing in an enhanced acoustic environment. In an early report by Clopton and Winfield [36], rats were exposed for the first 4 months of life to a 65 dB SPL frequency sweep in the 6–9 kHz range for an average of 5 hrs/day. Units in inferior colliculus were sampled. The greatest number of responses occurred between 4 and 5 kHz, that is, about 1 kHz below the lowest frequency of the exposure sweep. More recently, Oliver et al. [21] show increased representation of 13 kHz in adult rat IC, a frequency area lower than the neonatal exposure frequency of 14 kHz (a pure-tone delivered from P9 to P29 at 60–70 dB). Similarly, in a study in primary auditory cortex by Merzenich and colleagues [37], rats were exposed to a sequence of 30-ms 65-dB-SPL tone pips from P9 to P30. Here, the greatest percent change in cortical representation of adult rats was at 1.7 kHz, about an octave below the lowest tone stimulus presented. These studies provide evidence that developmental changes in tonotopic maps following neonatal exposure to tonal stimulation are often not limited to the exposure frequency, but that changes at low-frequency “edges” of the sound stimulus activation pattern may be a more consistent finding.

In summary, the data reported here support the hypothesis that persistent exposure to an abnormal sound environment during an early postnatal period can alter tonotopic organization in auditory midbrain. After prolonged neonatal exposure to a 2 ± 0.25 kHz signal, a significant overrepresentation of low frequencies (<1 kHz) was observed. Paradoxically, we found a decrease in the number of IC neurons tuned at or near 2 kHz. Our results suggest that in a precocious species (*chinchilla laniger*) similar to the human with regard to auditory development at birth, prolonged, abnormal neonatal sound exposure can result in neuroplastic change at the level of auditory midbrain.

## References

[1] Kraus H.-J., Aulbach-Kraus K. (1981). Morphological changes in the cochlea of the mouse after the onset of hearing. *Hearing Research*.

[2] Lim D. J. (1987). Development of the tectorial membrane. *Hearing Research*.

[3] Roth B., Bruns V. (1992). Postnatal development of the rat organ of Corti. I. General morphology, basilar membrane, tectorial membrane and border cells. *Anatomy and Embryology*.

[4] Roth B., Bruns V. (1992). Postnatal development of the rat organ of Corti-II. Hair cell receptors and their supporting elements. *Anatomy and Embryology*.

[5] Geal-Dor M., Freeman S., Li G., Sohmer H. (1993). Development of hearing in neonatal rats: air and bone conducted ABR thresholds. *Hearing Research*.

[6] Uziel A., Romand R., Marot M. (1981). Development of cochlear potentials in rats. *Audiology*.

[7] Pujol R., Lavigne-Rebillard M., Uziel A. (1991). Development of the human cochlea. *Acta Oto-Laryngologica*.

[8] Birnholz J. C., Benacerraf B. R. (1983). The development of human fetal hearing. *Science*.

[9] Querleu D., Renard X., Versyp F., Paris-Delrue L., Crèpin G. (1988). Fetal hearing. *European Journal of Obstetrics and Gynecology and Reproductive Biology*.

[10] Harrison R. V., Cullen J. R., Takeno S., Mount R. J. (1996). The neonatal chinchilla cochlea: morphological and functional study. *Scanning Microscopy*.

[11] Pienkowski M., Harrison R. V. (2005). Tone frequency maps and receptive fields in the developing chinchilla auditory cortex. *Journal of Neurophysiology*.

[12] Heffner R. S., Heffner H. E. (1991). Behavioral hearing range of the chinchilla. *Hearing Research*.

[13] Miller J. D. (1970). Audibility curve of the chinchilla. *Journal of the Acoustical Society of America*.

[14] Chang E. F., Merzenich M. M. (2003). Environmental noise retards auditory cortical development. *Science*.

[15] De Villers-Sidani E., Simpson K. L., Lu Y.-F., Lin R. C. S., Merzenich M. M. (2008). Manipulating critical period closure across different sectors of the primary auditory cortex. *Nature Neuroscience*.

[16] Norẽa A. J., Gourevich B., Aizawa N., Eggermont J. J. (2006). Spectrally enhanced acoustic environment disrupts frequency representation in cat auditory cortex. *Nature Neuroscience*.

[17] Stanton S. G., Harrison R. V. (1996). Abnormal cochleotopic organization in the auditory cortex of cats reared in a frequency augmented environment. *Auditory Neuroscience*.

[18] Stanton S. G., Harrison R. V. (2000). Projections from the medial geniculate body to primary auditory cortex in neonatally deafened cats. *Journal of Comparative Neurology*.

[19] Harrison R. V., Ibrahim D., Mount R. J. (1998). Plasticity of tonotopic maps in auditory midbrain following partial cochlear damage in the developing chinchilla. *Experimental Brain Research*.

[20] Yu X., Sanes D. H., Aristizabal O., Wadghiri Y. Z., Turnbull D. H. (2007). Large-scale reorganization of the tonotopic map in mouse auditory midbrain revealed by MRI. *Proceedings of the National Academy of Sciences of the United States of America*.

[21] Oliver D. L., Izquierdo M. A., Malmierca M. S. (2011). Persistent effects of early augmented acoustic environment on the auditory brainstem. *Neuroscience*.

[22] Brown T. A., Harrison R. V. (2010). Postnatal development of neuronal responses to frequency-modulated tones in chinchilla auditory cortex. *Brain Research*.

[23] Brown T. A., Harrison R. V. (2011). Neuronal responses in chinchilla auditory cortex after postnatal exposure to frequency-modulated tones. *Hearing Research*.

[24] Pienkowski M., Harrison R. V. (2005). Tone responses in core versus belt auditory cortex in the developing chinchilla. *Journal of Comparative Neurology*.

[25] Moore D. R., Aitkin L. M. (1975). Rearing in an acoustically unusual environment—effects on neural auditory responses. *Neuroscience Letters*.

[26] Poon P. W. F., Chen X. (1992). Postnatal exposure to tones alters the tuning characteristics of inferior collicular neurons in the rat. *Brain Research*.

[27] Sanes D. H., Constantine-Paton M. (1985). The sharpening of frequency tuning curves requires patterned activity during development in the mouse, Mus musculus. *Journal of Neuroscience*.

[28] Miyakawa A., Gibboni R., Bao S. (2013). Repeated exposure to a tone transiently alters spectral tuning bandwidth of neurons in the central nucleus of inferior colliculus in juvenile rats. *Neuroscience*.

[29] Schreiner C. E., Polley D. B. (2014). Auditory map plasticity: diversity in causes and consequences. *Current Opinion in Neurobiology*.

[30] Harrison R. V., Nagasawa A., Smith D. W., Stanton S., Mount R. J. (1991). Reorganization of auditory cortex after neonatal high frequency cochlear hearing loss. *Hearing Research*.

[31] Turrigiano G. G., Leslie K. R., Desai N. S., Rutherford L. C., Nelson S. B. (1998). Activity-dependent scaling of quantal amplitude in neocortical neurons. *Nature*.

[32] Kujawa S. G., Liberman M. C. (2009). Adding insult to injury: cochlear nerve degeneration after ‘temporary’ noise-induced hearing loss. *The Journal of Neuroscience*.

[33] Pienkowski M., Eggermont J. J. (2009). Long-term, partially-reversible reorganization of frequency tuning in mature cat primary auditory cortex can be induced by passive exposure to moderate-level sounds. *Hearing Research*.

[34] Pienkowski M., Eggermont J. J. (2010). Passive exposure of adult cats to moderate-level tone pip ensembles differentially decreases AI and AII responsiveness in the exposure frequency range. *Hearing Research*.

[35] Pienkowski M., Eggermont J. J. (2011). Cortical tonotopic map plasticity and behavior. *Neuroscience and Biobehavioral Reviews*.

[36] Clopton B. M., Winfield J. A. (1976). Effect of early exposure to patterned sound on unit activity in rat inferior colliculus. *Journal of Neurophysiology*.

[37] Nakahara H., Zhang L. I., Merzenich M. M. (2004). Specialization of primary auditory cortex processing by sound exposure in the ‘critical period’. *Proceedings of the National Academy of Sciences of the United States of America*.

